# Feasibility of a Single-Fraction Stereotactic Dose of 30 Gy to Solitary Lung Lesions on Halcyon

**DOI:** 10.7759/cureus.59535

**Published:** 2024-05-02

**Authors:** Joshua Misa, James A Knight, Damodar Pokhrel

**Affiliations:** 1 Department of Radiation Oncology, University of Kentucky, Lexington, USA

**Keywords:** lung lesion, vmat, single dose, sbrt, halcyon

## Abstract

Purpose

We sought to explore the feasibility of using the current co-planar Halcyon ring delivery system (RDS) with a novel multileaf collimator (MLC) aperture shape controller in delivering a single high dose of 30 Gy to solitary lung lesions via stereotactic body radiotherapy (SBRT).

Materials and methods

Thirteen non-small-cell lung cancer (NSCLC) patients previously treated with a single dose of 30 Gy to lung lesions via SBRT on the TrueBeam (6MV-FFF) using non-coplanar volumetric modulated arc therapy (VMAT) arcs were anonymized and replanned onto the Halcyon RDS (6MV-FFF) following RTOG-0915 single-fraction criteria. The Halcyon plans utilized a novel dynamic conformal arc (DCA)-based MLC-fitting approach before VMAT optimization with a user-defined aperture shape controller option. The clinical TrueBeam and Halcyon plans were compared via their protocol compliance, target conformity, gradient index, and dose to organs-at-risk (OAR). Treatment delivery efficacy and accuracy were assessed through end-to-end quality assurance (QA) tests on Halcyon and independent dose verification via in-house Monte Carlo (MC) second-check validation.

Results

All Halcyon lung SBRT plans met RTOG-0915 protocol’s requirements for target coverage, conformity, and gradient indices, and maximum dose 2 cm away from the target (D_2cm_) while being statistically insignificant (p > 0.05) when compared to clinical TrueBeam plans. Additionally, Halcyon provided a similar dose to OAR except for the ribs, where Halcyon demonstrated a lower maximum dose (15.22 Gy vs 17.01 Gy, p < 0.001). However, Halcyon plans required a higher total monitor unit (8892 MU vs 7413 MU, p < 0.001), resulting in a higher beam modulation factor (2.96 MU/cGy vs 2.47 MU/cGy, p < 0.001) and an increase in beam-on time by a factor of 2.1 (11.11 min vs 5.3 min, p < 0.005). End-to-end QA measurements demonstrate that Halcyon plans were clinically acceptable with an average gamma passing rate of 99.8% for 2%/2mm criteria and independent MC 2nd checks within ±2.86%.

Conclusion

Our end-to-end testing and validation study demonstrates that by utilizing a DCA-based MLC aperture shape controller before VMAT optimization, Halcyon can be used for delivering a single dose of lung SBRT treatment. However, future improvements of Halcyon RDS are recommended to allow higher output rates, rotational couch corrections, and an integrated intrafraction motion management system that will further enhance Halcyon’s capability for site-specific single dosage of SBRT.

## Introduction

Stereotactic body radiotherapy (SBRT) has become the standard of care approach for early-stage non-small cell lung cancer (NSCLC) patients. SBRT for NSCLC has demonstrated high local control rates, 92% for T1 tumors after five years [1], comparable to surgery while maintaining low rates of radiation-induced toxicities [1-6]. NSCLC can be treated by SBRT utilizing a variety of techniques including but not limited to volumetric modulated arc therapy (VMAT), 3D-conformal radiation therapy, tomotherapy, dynamic conformal arc (DCA) therapy, and robotic CyberKnife unit [7-11]. Furthermore, many studies have shown that a 6MV flattening filter-free (6MV-FFF) beam as opposed to a 6MV flattened beam for lung SBRT exhibited similar dosimetric results while offering lower treatment times which may potentially improve patient comfort, and compliance, and reduce intrafraction motion error [12-14].

Clinical studies investigating the utility of delivering single high-dose as opposed to multiple-fraction SBRT to solitary lung lesions have revealed comparable tumor local control while maintaining radiation-induced toxicities [15-17]. Single-fraction treatments are highly desirable as they improve patient compliance by being cost-effective and a non-surgical treatment option. For instance, Videtic et al. [16] investigated two SBRT schedules, 34 Gy in one fraction and 48 Gy in four fractions, for the treatment of selected early-stage peripheral NSCLC patients. They demonstrated that the single fraction treatments were able to attain similar tumor local control while achieving lower radiation-induced lung toxicity rates. Videtic et al. [17] further investigated this treatment approach in a prospective clinical trial by comparing two single fractions SBRT schedules, 30 Gy vs 34 Gy, and demonstrated that the 30 Gy scheme was statistically superior for toxicity profiles while providing similar tumor local control rate and overall survival. Based on these studies and our own clinical experience, our clinic has implemented a 30 Gy in 1 fraction treatment scheme for selected, medically inoperable, early-stage, peripherally located NSCLC patients.

Currently, there have been no reports investigating the use of the Halcyon ring delivery system (RDS) to deliver such high-stake lung SBRT treatments. Due to the required limited physics resources, Halcyon can be highly desirable to rural and single-linear accelerator (LINAC) clinics with relatively lower treatment costs and streamlined clinic workflow. Additionally, the Halcyon has been shown to have fewer downtimes and quickly recoverable faults that don’t require vendor field service engineers [18]. The Halcyon RDS is configured with a 6MV-FFF beam with a maximum output of 800 MU/min which is notably lower than the corresponding TrueBeam’s 6MV-FFF maximum dose rate of 1400 MU/min. Although limited by its three degrees-of-freedom (3DoF) couch correction compared to TrueBeam’s 6DoF PerfectPitch couch, it is capable of reproducing isocenter positional accuracy similar to the TrueBeam LINAC [19] and was well within tolerance for SBRT treatments as recommended by TG-101 [20]. Varian Eclipse Treatment Planning System (TPS) (Version 16.2, Varian Medical Systems, Palo Alto, United States) has recently implemented a multileaf collimator (MLC) aperture shaper controller photon optimizer (PO) on Halcyon RDS where Eclipse users can control the MLC field aperture shape and calculate a 3D dose distribution via DCA approach before VMAT optimization on Halcyon. Thus, as a part of our Halcyon commissioning and validation of Eclipse TPS (Version 16.2), it was important for us to highlight the importance of investigating the new treatment planning features in Eclipse. This study aims to assess the feasibility and accuracy of delivering 30 Gy single fraction lung SBRT treatments on the jawless, single energy Halcyon V2.0 unit utilizing an MLC aperture shaper controller and benchmarking with clinical non-coplanar TrueBeam lung SBRT treatments.

## Materials and methods

Patient characteristics, CT imaging, and target delineation

After obtaining approval from the University of Kentucky, Institutional Review Board (approval no. 92667), the study was conducted at the University of Kentucky, Lexington, United States. Thirteen early-stage I-II NSCLC patients who previously underwent lung SBRT treatment of 30 Gy in one fraction of peripherally located lung tumors were identified and anonymized for this benchmarking study. These patients were selected based on guidelines outlined by the RTOG-0915 protocol. All clinical treatments were delivered on a Varian TrueBeam LINAC.

During CT simulation, all patients were immobilized using Body Pro-LokTM (CIVCO RT, Avondale, United States) while positioned supine with their arms above their heads. First, a free-breathing CT scan was acquired with a 2.0 mm slice thickness followed by a 4D-CT scan using the Varian RPM System (version 1.7). The 4D-CT images were used to generate a maximum intensity projection (MIP) scan. For target delineation, an internal target volume (ITV) was contoured using the 4D-MIP co-registered with the free-breathing CT scan. The planning target volume (PTV) consists of the ITV with a 5-8 mm uniform margin expansion per physicians’ discretion. The tumor characteristics including location and size for all cases included are summarized in Table 1. The critical organs-at-risks (OARs) were contoured on free-breathing CT images and consisted of the bilateral lungs excluding ITV (healthy lung), spinal cord, heart, esophagus, trachea, ribs, and skin.

**Table 1:** Patient characteristics for single fraction 30 Gy validation study on Halcyon. Patients were originally treated on a TrueBeam via non-coplanar arc geometry ITV: internal target volume; PTV: planning target volume; cc: cubic centimeter; SD: standard deviation; Gy: gray

Patient #	ITV (cc)	PTV (cc)	Tumor location
1	2.4	17.7	Left upper lobe
2	7.6	26.0	Left upper lobe
3	4.7	17.2	Right upper lobe
4	3.7	14.4	Right medial lobe
5	3.9	14.3	Left upper lobe
6	1.2	12.3	Right upper lobe
7	1.8	13.4	Right upper lobe
8	0.4	6.5	Right lower lobe
9	1.0	9.7	Right upper lobe
10	0.3	4.3	Left upper lobe
11	6.6	29.5	Right medial lobe
12	0.8	11.4	Left upper lobe
13	1.0	12.8	Left lower lobe
Mean ± SD (range)	2.96 ± 2.32 (0.4-7.6)	14.40 ± 7.58 (4.36-29.7)	

Treatment planning

All clinical lung SBRT plans included in this study were planned with Eclipse TPS version 15.6 using 2-6 unilateral partial non-coplanar VMAT arcs (±5-10^o^ couch kicks) with 6MV-FFF beam (1400 MU/min). Treatments were delivered on Varian’s TrueBeam LINAC with standard millennium 120MLC via pretreatment conebeam CT guidance. These plans utilized patient-specific collimator rotations and had jaw tracking enabled. All cases were then retrospectively replanned using Eclipse version 16.2 by an experienced SBRT physicist and delivered on the Halcyon RDS for end-to-end quality assurance (QA). These Halcyon SBRT plans utilized six unilateral partial coplanar VMAT arcs mimicking TrueBeam’s arc length and collimator settings. During VMAT optimization, the MU objective was used with a maximum monitor unit limit of 8000 MU. To obtain a similar plan quality as clinical TrueBeam plans, different optimization objectives were used on Halcyon. Both Truebeam and Halcyon plans before VMAT optimization had their MLCs fit to the PTV structure and had an initial DCA-based 3D dose distribution calculated. Both sets of plans used an MLC aperture shape controller with high priority. Additionally, both sets of plans utilized Eclipse’s advanced Acuros-based dose calculation models with a fine resolution (1.25 mm) dose calculation grid size with tissue heterogeneity corrections turned on. All physics QA including machine QA tests and patient-specific QA processes were followed per recommendations laid out by the AAPM TG-101 protocol [20].

Plan evaluation

The Halcyon lung SBRT plans were dosimetrically compared to clinical plans via conformity index (CI = ratio of prescribed isodose volume to PTV volume); Paddick confirmation number [21] (PCN = TV_PIV_^2^ ÷ (TV × PIV), where TV = treated volume and PIV = prescription isodose volume); gradient index (GI = ratio of 50% isodose volume to PTV volume); intermediate dose spillage (D2cm = maximum dose 2 cm away from the PTV); and the gradient measure (GM, difference of equivalent sphere radii of the 50% and 100% prescription volumes). Dose to adjacent critical organs was evaluated using RTOG-0915 protocol guidelines (Arm 1) and TG-101 recommendations of a single-dose scheme for all plans [20]. Furthermore, treatment delivery efficiency was compared with each plan’s total monitor units (MU), modulation factor (MF = ratio of the total MU to the prescription dose in cGy), and beam-on-time (BOT). Treatment delivery accuracy and verification of the Halcyon SBRT plans were performed via routine portal dosimetry quality assurance (PD-QA) using our clinical gamma criteria of 2%/2mm with a low-dose threshold set to 10%. These PD-QAs were performed with the electronic portal imaging device (EPID, aS1200 flat panel detector, Varian Medical Systems, Palo Alto, United States) mounted on the Halcyon RDS. Additionally, independent dose verification of Halcyon’s lung SBRT plans was performed via in-house Monte Carlo (MC) calculation as part of our clinical physics second check procedure. Statistical analysis was performed using a paired samples t-test with a statistically significant ­p-value to be less than 0.05.

## Results

Target coverage and intermediate dose spillage

All clinical TrueBeam and Halcyon SBRT plans were acceptable based on RTOG-0915 requirements. Table 2 summarizes the results comparing target conformity and intermediate dose fall-off. In comparison to the clinical plans, Halcyon demonstrated similar target conformity despite being limited to coplanar beam geometry. Analysis of the intermediate dose fall-off of Halcyon plans shows that it achieved similar GI and D2cm to clinical TrueBeam plans while demonstrating a statistically significant improvement to GM. It should be noted that GM is not a parameter included in RTOG-0915 to be evaluated for these types of treatment plans.

**Table 2:** Evaluation of plan quality metrics for target coverage and intermediate dose spillage for a single dose of 30 Gy lung SBRT treatment on Halcyon, compared to clinical TrueBeam plans Mean ± SD (range) was reported for all parameters. The statistically significant p-value is highlighted in bold. PTV: planning target volume; CI: conformity index; PCN: Paddick confirmation number; GI: gradient index; GM: gradient measure; Gy: gray; SD: standard deviation; D2cm: maximum dose 2cm away from PTV; SBRT: stereotactic body radiotherapy

Target volume	Parameters	Clinical plans	Halcyon plans	p-­value
PTV coverage and Intermediate dose spillage	CI	1.02 ± 0.03 (0.98-1.09)	1.00 ± 0.04 (0.90-1.08)	0.289
	PCN	0.89 ± 0.03 (0.83-0.94)	0.91 ± 0.04 (0.81-0.96)	0.122
	GI	4.67 ± 0.58 (3.87-5.97)	4.55 ± 0.71 (3.76-6.43)	0.232
	GM (cm)	0.99 ± 0.11 (0.79-1.13)	0.95 ± 0.09 (0.83-1.08)	0.040
	D_2cm _(%)	48.76 ± 2.46 (44.90-53.80)	48.43 ± 3.15 (42.60-53.70)	0.589

**Figure 1:**
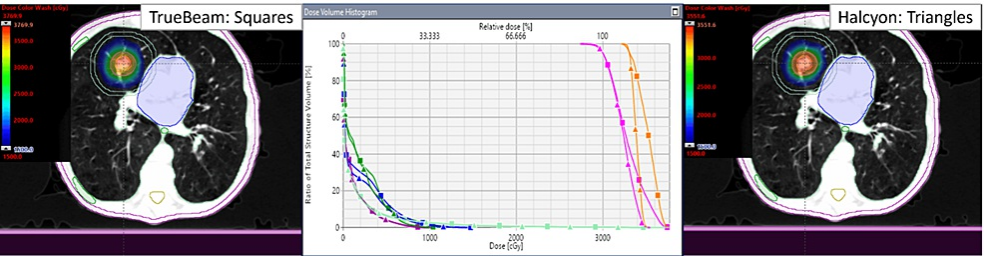
Isodose colorwash distributions for clinical TrueBeam (left) and replanned Halcyon plan (right)-crosshair shows the isocenter location, and DVH parameters (center). For a 14.4 cc right medial lobe tumor of PTV (pink) and ITV (orange), improvements to CI, GI, PCN, D2cm, and GM were obtained with Halcyon. Adjacent critical structure’s DVH shown are the ribs (green), normal lung (sky blue), heart (dark blue), and skin (light brown). D2cm ring (cyan) is also shown Truebeam: Squares; Halcyon: Triangles. DVH: dose-volume histogram; cc: cubic centimeter; PTV: planning target volume; ITV: internal target volume; CI: conformity index; GI: gradient index; GM: gradient measure; SD: standard deviation; D2cm­: maximum dose 2cm away from PTV; cGy: centigray

Figure 1 depicts an example case (patient #2) comparing the clinical TrueBeam plan vs Halcyon replan. The Halcyon replan was able to achieve an improved CI (0.99 vs 1.06), PCN (0.93 vs 0.85), GI (4.79 vs 5.15), GM (1.03 cm vs 1.12 cm), and D2cm (46.5% vs 49.8%) compared to the clinical TrueBeam plan. In terms of healthy lungs’ V20Gy and V10Gy parameters, this Halcyon plan had slightly higher values compared to its corresponding TrueBeam plan, 0.73% vs. 0.70% and 4.4% vs. 2.8%, respectively. However, both plans provided significantly lower V20Gy as required by RTOG-0915 (acceptable <10%). Halcyon plans gave lower maximum doses to the ribs (10.1 Gy vs 12.1 Gy) and the heart (14.4 Gy vs 15.6 Gy), but a slight increase in dose to the skin (10.3 Gy vs 10.0 Gy) when compared to non-coplanar-TrueBeam-SBRT plan. In terms of delivery efficiency, this Halcyon plan required a longer BOT of 9.78 min as compared to the clinical TrueBeam plan’s BOT of 5.18 min.

Dose to critical organs

While following the RTOG-0915 requirements, both lung SBRT plans were within compliance with OAR constraints outlined by the RTOG-0915 protocol for a single-fraction lung treatment scheme. A summary of the OAR and the dose metrics analyzed is shown in Table 3. Even with coplanar geometry, while utilizing the novel DCA-based approach, the Halcyon VMAT plans were able to achieve statistically similar maximum point dose and volumetric dose parameters for the spinal cord, heart, esophagus, trachea, skin, and healthy lung. The only exception was the ribs, in which the Halcyon showed a statistically significant decrease in maximum point dose and dose to 1 cc of ribs, with an average reduction of 1.8 Gy and 1.6 Gy, respectively.

**Table 3:** Evaluation of dose to adjacent OAR for a single dose of 30 Gy lung SBRT re-planned on Halcyon compared to clinical TrueBeam plans Mean ± SD (range) was reported for all evaluated parameters. Statistically significant p-values are highlighted in bold. SD: standard deviation; OAR: organ at risk; D_max_: maximum dose; D_Xcc_: minimum dose covering at least X cc of the target volume; V_XGy_: volume receiving X Gy; SBRT: stereotactic body radiotherapy; Gy: gray

OAR volume	Parameters	Clinical plans	Halcyon plans	p-­value
Spinal cord	D_max_ (Gy)	4.03 ± 1.99 (1.27-7.28)	3.96 ± 1.77 (1.34-6.20)	0.548
	D_0.35cc _(Gy)	3.47 ± 1.62 (1.08-5.94)	3.51 ± 1.55 (1.28-5.57)	0.642
Heart	D_max_ (Gy)	6.86 ± 5.31 (0.18-15.56)	6.18 ± 5.17 (0.20-14.39)	0.086
	D_15cc _(Gy)	3.34 ± 2.80 (0.11-9.04)	3.65 ± 3.01 (0.13-8.37)	0.417
Esophagus	D_max_ (Gy)	5.77 ± 3.47 (2.41-15.32)	5.42 ± 2.75 (2.29-11.91)	0.239
	D_5cc _(Gy)	2.25 ± 1.11 (0.19-3.75)	2.10 ± 1.23 (0.18-4.65)	0.55
Trachea	D_max_ (Gy)	4.17 ± 4.73 (0.04-13.67)	3.91 ± 4.02 (0.05-12.10)	0.475
	D_4cc _(Gy)	1.28 ± 1.58 (0.02-4.82)	1.12 ± 1.54 (0.03-4.69)	0.497
Ribs	D_max_ (Gy)	17.01 ± 5.36 (10.15-28.66)	15.22 ± 5.04 (9.18-26.06)	<0.001
	D_1cc _(Gy)	13.37 ± 3.09 (8.97-17.22)	11.82 ± 3.56 (6.62-17.42)	<0.001
Skin	D_max_ (Gy)	8.84 ± 1.61 (5.71-10.87)	8.70 ± 1.11 (6.82-10.43)	0.595
	D_10cc _(Gy)	4.51 ± 1.50 (0.22-6.42)	5.45 ± 0.82 (4.45-7.31)	0.093
Healthy lung	V_20Gy_ (%)	0.60 ± 0.29 (0.22-1.25)	0.56 ± 0.26 (0.19-1.11)	0.055
	V_10Gy_ (%)	2.62 ± 1.11 (1.10-4.76)	2.64 ± 1.14 (1.09-4.50)	0.867

Treatment delivery and accuracy

Analysis of both PD QA results and independent MC 2nd check calculations show that Halcyon RDS can deliver these types of treatments with clinically acceptable accuracy. On average, the Halcyon plans had a gamma passing rate of 99.8% and MC 2nd check results showed that Eclipse’s TPS calculation was accurate within ±2.86%. The MC 2nd check results were statistically lower for the Halcyon plans even though both platforms used a DCA-VMAT approach. This result may be due to the increase in beam modulation required for the Halcyon plans and due to the MC model’s less accurate modeling of the stack and staggered Halcyon MLCs. Head-to-head comparisons of treatment delivery metrics between the two sets of plans reveal many inadequacies with the current Halcyon LINAC as summarized in Table 4.

**Table 4:** Comparison to clinical TrueBeam plans, evaluation of delivery efficiency for a single dose of 30 Gy lung SBRT treatment on Halcyon in comparison to clinical TrueBeam plans Mean ± SD (range) was reported for all parameters. Statistically significant p-values are highlighted in bold. MF: modulation factor; SD: standard deviation; MC: Monte Carlo; MU: monitor units; Gy: gray; cGy: centigray; SBRT: stereotactic body radiotherapy

Delivery parameters	Clinical plans	Halcyon plans	p-value
Total monitor units (MU)	7413 ± 1580 (5533-10869)	8891.8 ± 1673.2 (5935.9-11273.1)	<0.001
MF (MU/cGy)	2.47 ± 0.53 (1.84-3.62)	2.96 ± 0.56 (1.98-3.76)	<0.001
Beam-on time (minutes)	5.29 ± 1.13 (3.95-7.76)	11.12 ± 2.09 (7.42-14.09)	<0.001
Independent MC dose verification (%)	1.03 ± 1.56 (-0.80-4.00)	2.86 ± 1.60 (0.00.7-5.60)	<0.001

First, the Halcyon lung SBRT plans required on average 20% more total MU than corresponding TrueBeam plans which results in increased beam MF by a factor of 1.2. Due to the Halcyon’s lower maximum dose rate of 800 MU/min compared to TrueBeam’s corresponding dose rate of 1400 MU/min in combination with having 1.2 times higher total MU, the Halcyon plans averaged a relatively longer BOT by a factor of 2.1 in this setting.

## Discussion

In this technical report, we validated and benchmarked the performance of single high-dose lung SBRT treatments on the Halcyon RDS while following RTOG-0915 single fraction protocol requirements. Patient selection criteria followed RTOG-0915 protocol (Arm 1), where patients present with solitary, peripherally located lung lesions less than 4 cm in diameter (and no adjacent OAR) can be planned on Halcyon RDS. Utilizing the new Eclipse planning feature of aperture shape controller with high priority and DCA-based VMAT approach, Halcyon has demonstrated dosimetrically similar target conformity and dose gradient to clinical TrueBeam plans despite being limited to coplanar beam geometry. Furthermore, the Halcyon plans were in compliance with RTOG-0915 constraints for protecting adjacent critical organs while providing a reduction to ribs dose in comparison to clinical TrueBeam plans. Additionally, the treatment delivery accuracy of the Halcyon SBRT plans was clinically acceptable with PD-QA measurements using a gamma criterion of 2%/2mm showing average passing rates of 99.8% and independent MC-based dose verification calculations being within ±2.86%.

However, there are several downsides to utilizing the current Halcyon RDS for these types of high-stake single-dose treatment schemes. One such drawback is the significant increase in total MU required for the Halcyon. We observed in this setting, that the Halcyon plans required on average 1479 more MU than clinical TrueBeam plans. The increase in MU plus the lower maximum output on the Halcyon compared to the TrueBeam leads to Halcyon plans to require a BOT of 2.1 times longer than clinical TrueBeam plans. The longer BOT from the Halcyon plans may be concerning for lung SBRT patients with shortness of breath and back pain, potentially decreasing patient compliance and comfort in addition to an increased risk of intra-fractional motion error. Another potential concern with these highly modulated SBRT plans with relatively long delivery times for Halcyon plans, is a potential machine overheating issue. Two Halcyon plans that required over 14 min of BOT resulted in water overheating cooling interlocks engaged during patient-specific QA; however, we were able to acknowledge the machine cooling interlock and continue delivering all monitor units. However, in actual SBRT treatment, these interruptions during treatment delivery may further aggravate patient comfort while further increasing the risk of significant intra-fractional motion error occurring. The overheating interlock occurred around 10,000 MUs, so we suggest precautions be taken for Halcyon SBRT plans requiring more than 10,000 MU. One should be wary of the already elevated machine temperatures during a typical clinical workday when scheduling these types of high-stake single-dose treatments. Future work will involve performing a full FMEA-style analysis of Halcyon's ability to deliver these treatments. Another limitation is Halcyon’s lack of output factors for field sizes smaller than 1.0 cm × 1.0 cm, suggesting lung SBRT targets <1 cm in diameter have a major concern of small field dosimetry errors during treatment planning and delivery on the Halcyon [22]. Thus, for the Halcyon system, as it is now, we do not recommend its use for single high-dose treatments for small targets unless the vendor implements significant upgrades in the future providing these small field output factors.

Herein we propose multiple recommendations to the vendor for potential future improvements to be implemented on the Halcyon RDS. First, we suggest that Halcyon’s maximum dose rate be tuned up to be similar to TrueBeam. For example, if Halcyon has a dose rate of 1000 MU/min, these SBRT plans can be delivered within an average BOT of 8.9 min. If tuned up to TrueBeam’s maximum corresponding dose rate of 1400 MU/min (6MV-FFF), the Halcyon should be able to deliver these plans in 6.4 min, like the clinical TrueBeam plans even with higher beam modulation. Second, the incorporation of an ARIA-based online intra-fraction motion management system is highly recommended. With Halcyon’s longer BOT, to limit the error of intra-fractional motion issues, an online motion management system is of utmost importance for a single dose of lung SBRT. For select patients, this may also allow for deep-inspiration breath hold (DIBH) lung SBRT in the future. Third, the development and implementation of the rotational couch corrections method will significantly improve treatment delivery accuracy of the lung SBRT plans on Halcyon; however, as of now, we do not know exactly how much improvement this could make in the area of future investigation. With these potential upgrades, Halcyon may treat patients faster than TrueBeam plans due to the implemented fully automated “one-step patient set-up and verification” method on Halcyon. As discussed before, other potential recommendations we provide are to improve the machine cooling capabilities to allow Halcyon plans requiring >10,000 MU to be delivered with no interruptions and to expand the output factors table to include field sizes smaller than 1.0 cm × 1.0 cm to allow for more accurate treatment planning and delivery of small targets.

A summary of our recommendations to the vendor is shown in Table 5. If implemented, the Halcyon will become a more attractive treatment unit for rural communities and single-LINAC clinics allowing for this treatment technique to be more widely implemented in the future. Moreover, with these improvements Halcyon can be used to transfer patients for longer machine downtime of TrueBeam, resulting in significant treatment course delays in the academic centers or a busy SBRT program. These upgrades will allow for safely and effectively delivering a single dose of 30 Gy lung SBRT treatments on Halcyon and expand the use of high-quality, stereotactic lung SBRT treatment programs to the community centers for underserved patient cohorts.

**Table 5:** Our recommendations to the vendor for further improvement of Halcyon; which may allow for routine stereotactic treatment of a single high dose of SBRT including lung lesions SBRT: stereotactic body radiotherapy; MU: monitor unit; 4D-iCBCT: 4D iterative cone-beam computed tomography; 6DOF: 6 degrees of freedom

Current Halcyon limitations for single high dose of SBRT treatments	Our recommendations
Long beam-on times	Tune up dose rate to 1000MU/min, possibly to 1400MU/min
Intra-fraction motion monitoring	Develop ARIA-based online motion management protocol with 4D-iCBCT scanning capability
Patient set up accuracy	Introduce rotational couch corrections via 6DOF
Machine overheating issue	Improve machine cooling system including target cooling
Small fields output factor	Provide output factors down to 0.5 × 0.5 cm^2^ field size in Eclipse

The future of the Halcyon is for it to be able to deliver single fraction SBRT treatments for a variety of treatment sites, safely and effectively. For single fraction SBRT treatments to the lung lesion, Chuong et al. [23] have demonstrated that MRI-LINACs are able to deliver such treatments safely and within a reasonable amount of time. We predict that the Halcyon should be able to dosimetrically outperform the MRI-LINAC system, even though they are both limited to coplanar geometry. As of now, the MRI-LINAC is further limited by not having the ability to deliver VMAT plans; static IMRT delivers higher MU compared to VMAT plans. Another future treatment Halcyon should aim to perform is a single fraction of SBRT cardiac ventricular tachycardia (VT) treatments. Recently, there has been increased clinical implementation of treating VT via SBRT with a prescription dose of 25 Gy in a single fraction. Cardiac VT SBRT plans can be effectively delivered using coplanar geometry and with great clinical outcomes [24]. Thus we should expect that Halcyon plans could be dosimetrically acceptable for this prescription in the future as well. Again, the concern is long treatment times because of the high required MU for Halcyon plans as well as its relatively lower maximum dose rate compared to typical C-arm LINACs with FFF beams such as the TrueBeam. Last, Halcyon RDS may be a viable treatment unit for delivering single-fraction SBRT treatment for locally advanced pancreatic cancers in the future. Koong et al. [25] showed that on the robotic CyberKnife platform, a single dose of 25 Gy via SBRT for pancreatic cancer resulted in a very high local control rate of 94% while maintaining low rates of toxicities. As described above, a hurdle the current Halcyon faces is the lack of an online intrafraction motion management system that will be adequate enough to perform pancreatic treatments. Future development of a gating system on the Halcyon will be able to make pancreatic SBRT deliverability possible. We predict that the Halcyon RDS with the future improvements as recommended above would be a viable complement to other complex delivery systems in delivering these high-stake single fraction SBRT treatments for a wide range of disease types.

## Conclusions

We have investigated the feasibility and accuracy of utilizing the novel MLC aperture shape controller on Halcyon RDS plans DCA-based VMAT method and found that it can be used for selected patients for a single dose of lung SBRT treatment compliant with RTOG-0915 protocol if required. However, several future improvements of Halcyon RDS are suggested including higher output rates, rotational couch corrections, small field output factors, superior machine cooling system, and integrated online intrafraction motion management system that will further enhance Halcyon’s capability for stereotactic lung SBRT, including site-specific single dose of SBRT treatments in the remote and underserved centers in the future.
